# Protocol for the prediction, interpretation, and mutation evaluation of post-translational modification using MIND-S

**DOI:** 10.1016/j.xpro.2023.102682

**Published:** 2023-11-18

**Authors:** Yu Yan, Dean Wang, Ruiqi Xin, Raine A. Soriano, Dominic C.M. Ng, Wei Wang, Peipei Ping

**Affiliations:** 1 NIH BRIDGE2AI Center at UCLA & NHLBI Integrated Cardiovascular Data Science Training Program at UCLA, Suite 1-609, MRL Building, 675 Charles E. Young Dr. South, Los Angeles, CA 90095-1760, USA; 2 Medical Informatics Program, University of California at Los Angeles (UCLA), Los Angeles, CA 90095, USA; 3 Department of Physiology, UCLA School of Medicine, Suite 1-609, MRL Building, 675 Charles E. Young Dr., Los Angeles, CA 90095-1760, USA; 4 Scalable Analytics Institute (ScAi) at Department of Computer Science, UCLA School of Engineering, Los Angeles, CA 90095, USA; 5 Department of Medicine (Cardiology), UCLA School of Medicine, Suite 1-609, MRL Building, 675 Charles E. Young Dr. South, Los Angeles, CA 90095-1760, USA; 6 Computational and Systems Biology Interdepartmental Program (IDP), University of California at Los Angeles (UCLA), Los Angeles, CA 90095, USA; 7 Department of Computer Science, UCLA School of Engineering, Los Angeles, CA 90095, USA

**Keywords:** Bioinformatics, Sequence Analysis, Protein Biochemistry, Computer Sciences

## Abstract

Post-translational modifications (PTMs) serve as key regulatory mechanisms in various cellular processes; altered PTMs can potentially lead to human diseases. We present a protocol for using MIND-S (multi-label interpretable deep-learning approach for PTM prediction-structure version), to study PTMs. This protocol consists of step-by-step guide and includes three key applications of MIND-S: PTM predictions based on protein sequences, important amino acids identification, and elucidation of altered PTM landscape resulting from molecular mutations.

For complete details on the use and execution of this protocol, please refer to Yan et al (2023).^1^

## Highlights

• A protocol on MIND-S, a software program that supports multiple computational analyses on PTMs
• Steps described for performing multi-protein multi-PTM prediction
• Evaluation of important amino acids for PTM occurrences
• Examination of the SNP effect on PTMs

## Abstract (editor highlights)

Publisher’s note: Undertaking any experimental protocol requires adherence to local institutional guidelines for laboratory safety and ethics.

## Graphical abstract

**Figure:**
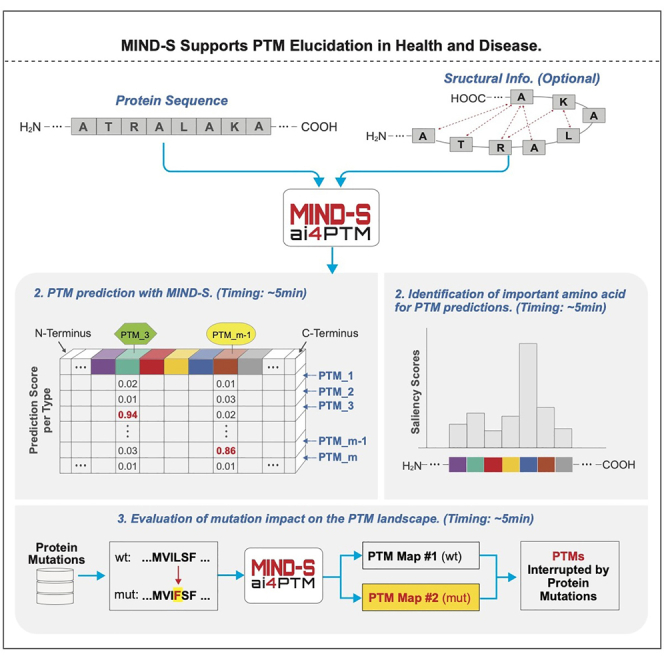

## Before you begin

Most protein post-translational modifications (PTMs) involve covalent processing events that alter the biophysical properties of a protein through the addition of a modifying group to one or more amino acids.^2^ PTMs often serve as key regulatory mechanisms governing a broad spectrum of sub-proteomes and are frequently involved in disease phenotypes.^3^^,^^4^ Publicly available PTM databases and advancements in artificial intelligence (AI) have prompted the development of fast and cost-efficient computational methods for large-scale PTM prediction.^3^^,^^4^^,^^5^^,^^6^ MIND-S (multi-label interpretable deep-learning method for PTM prediction-structure version) offers a unique workflow from the deep-learning models for large-scale multi-PTMs prediction.^1^ MIND-S is a neural network model with an architecture consisting of one embedding layer, one bidirectional LSTM layer, three multi-head self-attention blocks, one graph attention layer and one fully connected layer. MIND-S was trained on more than 40,000 proteins, encompassing more than 210,000 PTMs across 26 different types,^6^ with 13 types of curated PTMs and 13 oxidative PTMs (O-PTMs). MIND-S takes protein sequence and structure as input to generate a prediction of PTM at every target residue for 26 types of PTMs. At the core of the model there are three multi-head self-attention blocks and a graph neural network, responsible for sequence and structure, respectively. MIND-S also features interpretations on the prediction and evaluation of the impact of single nucleotide polymorphism (SNP) on PTM. MIND-S uses integrated gradients to evaluate feature importance, which indicates the relative contribution of flanking amino acids to the prediction. MIND-S can also be performed to study the effect of SNP in PTM by comparing prediction scores between wild-type protein and mutant protein.

In this protocol, we utilize one protein, Leucine-rich repeat serine/threonine-protein kinase 2 (*LRRK2*, UniProt: Q5S007), as an example to illustrate the three applications of MIND-S: predicting 26 types of PTMs using the full protein sequence as the input; identifying important amino acids of a specific PTM; and evaluating how mutation will alter the PTM landscape of this protein. Using the same strategy, one can investigate many other proteins and their PTMs of interest.

### Prepare input data

**Timing: 5 min**

1. Prepare protein sequence as a fasta file. Optionally, follow the steps below to download it from UniProt (Figure S1):
  a. Navigate to https://www.uniprot.org/id-mapping.
  b. Enter UniProt ID (e.g., UniProt: Q5S007) into the input field and select ‘Map IDs.’
  c. Wait for the job to complete, then click ‘Completed’ under the ‘Status’ column.
  d. Select ‘Download’.
  e. Change ‘Format’ to ‘FASTA (canonical),’ and under ‘Compressed’ select ‘No,’ and select ‘Download’.
  f. Save the file and rename it to *Q5S007.fasta.*

***Note:*** A list of UniProt accession IDs can be entered into the input field to perform batch sequence download.

2. Prepare protein mutation of interest.
  a. MIND-S requires the information of mutant amino acid location, wild-type amino acid, and mutant amino acid. For our example, we will examine the effects of a mutation that mutates the arginine at site 1441 to cysteine.

***Note:*** We recommend using Ensembl Variant Effect Predictor (VEP) to convert mutations on the genomic level to the protein level. VEP has an online graphical user interface as well as an API for programmatic access. It accepts inputs in the form of a Variant Call Format (vcf) file or SNP id and outputs detailed information on the protein mutations, if they exist.^7^

***Note:*** MIND-S allows users to examine mutation effects on PTM predictions. Here we provide a script for single amino acid mutation. Other mutations can be analyzed similarly.

### Set up the environment for running the program

**Timing: within 2 h**

3. Follow the instructions to install tensorflow2 (https://www.tensorflow.org/install/pip), if not already installed.
4. Clone the MIND-S repo from GitHub.

>git clone https://github.com/yuyanislearning/MIND.git

5. Change the directory to the newly created folder.

>cd MIND

6. Install the required Python packages indicated in the requirement file.

>pip install -r requirement

***Note:*** This protocol has been verified to run successfully in the following environment: Ubuntu 20.04 with a Tesla T4 GPU and CUDA version 12.0, python version 3.10, TensorFlow 2.11.0.^8^

## Key resources table

**Table:** 

REAGENT or RESOURCE		SOURCE	IDENTIFIER
**Software and algorithms**			

MIND-S	GitHub		https://github.com/yuyanislearning/MIND
Python 3.10	Python software foundation		https://www.python.org
Tensorflow 2.11.0	Google		https://www.tensorflow.org/
MIND-S protocol	Zenodo		https://doi.org/10.5281/zenodo.8393338

## Step-by-step method details

### PTM prediction with MIND-S

**Timing: 5 min**

Given a protein sequence (accepted in fasta format), MIND-S will predict if PTMs will occur on the protein, with detailed information about the PTM type and the PTM site. Predictions will be made on all targeted amino acids in the protein.

1. Specify an output directory to store the output.

>mkdir result

2. Run the following to make predictions.

>python batch_predict.py \

> --pretrain_name saved_model/MIND_fifteenfold \

> --data_path sample/Q5S007.fa \

> --res_path result \

> --n_fold 15

***Note:*** The trained model is stored in the saved_model directory (downloaded along with the MIND-S program) and can be specified by the pretrain_name parameter. We recommend using the MIND_fifteenfold model for predicting the 13 PTM types with n_fold = 15 for its most robust performance, achieved by repeating the training process 15 times. For predicting the 13 O-PTM types, set pretrain_name to OPTM_fifthteen. The data_path argument is used to provide the path to the protein fasta file and res_path is used to provide the path to the output folder, where the model outputs will be stored.

**Figure 1:**
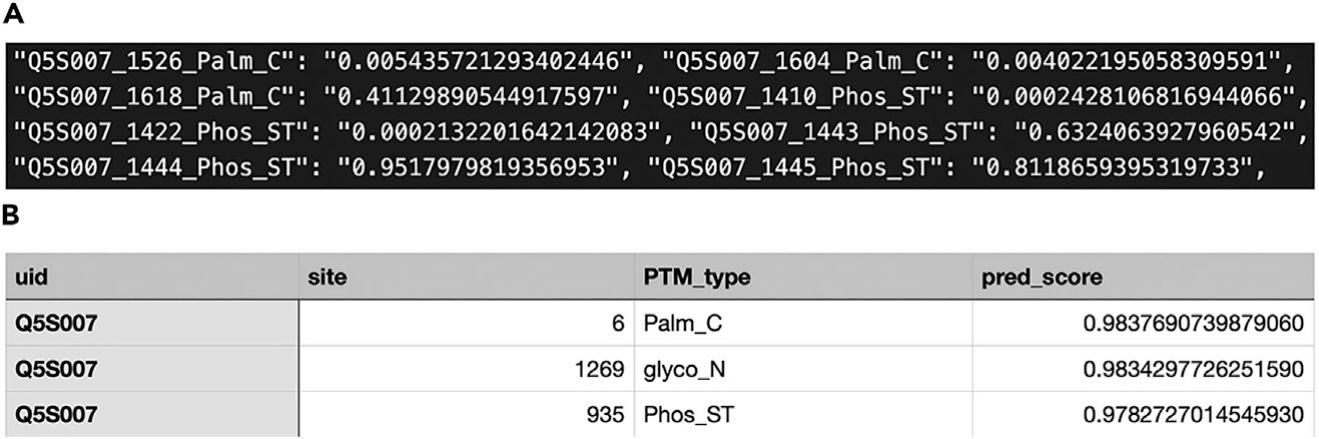
Example PTM prediction output files (A) screenshot from “results.json”. Each prediction is labeled as <protein UID_PTM site_PTM type: prediction score>. (B) screenshot from ‘correct_predictions.csv.’ The columns correspond to protein UID, PTM site, PTM type, and prediction score.

**Figure 2:**
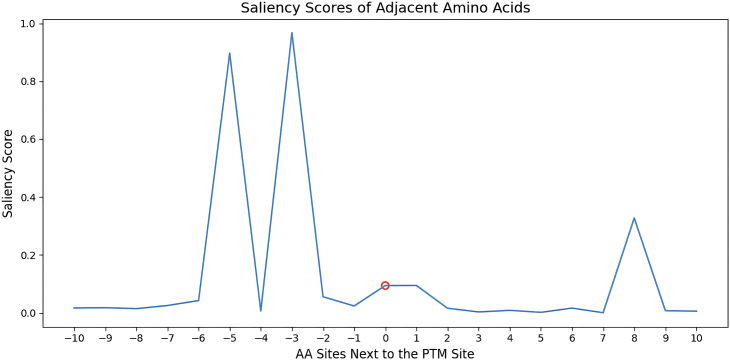
Saliency Score Plot of Amino Acids Spanning the PTM Site The x-axis refers to the relative positions of amino acids surrounding the PTM site, where 0 denotes the PTM site (a small red circle highlights the position of the PTM site). The y-axis refers to the saliency scores of the corresponding amino acids. A high saliency score indicates that the amino acid at the position is important for the model to make the prediction. In this case, amino acids at positions -3 and -5 are important for the prediction.

3. Examine the results.
  a. Two files, ‘results.json’ and ‘correct_predictions.csv’, will be returned from MIND-S under the output folder (Figures 1A and 2B).

**Table 1:** PTM and O-PTM type abbreviations

PTM type	Targeted amino acid	Abbreviation
**PTMs**		

Hydroxylysine (K)	K	Hydro_K
Hydroxyproline (P)	P	Hydro_P
Methylation (K)	K	Methy_K
Methylation (R)	R	Methy_R
N6-acetyllysine (K)	K	N6-ace_K
S-Palmitoylation (C)	C	Palm_C
Phosphorylation (ST)	S or T	Phos_ST
Phosphorylation (Y)	Y	Phos_Y
Pyrrolidone-carboxylic-acid (Q)	Q	Pyro_Q
SUMOylation (K)	K	SUMO_K
Ubiquitin (K)	K	Ubi_K
N-linked glycosylation (N)	N	glyco_N
O-linked glycosylation (ST)	S or T	glyco_ST

**O-PTMs**		

Arginine hydroxylation (R)	R	Arg-OH_R
Asparagine hydroxylation (N)	N	Asn-OH_N
Aspartate hydroxylation (D)	D	Asp-OH_D
Cysteine 4-HNE (C)	C	Cys4HNE_C
Cysteine sulfination (C)	C	CysSO2H_C
Cysteine sulfonation (C)	C	CysSO3H_C
Lysine carbonylation (K)	K	Lys2AAA_K
Methionine sulfonation (M)	M	MetO2_M
Methionine sulfoxide (M)	M	MetO_M
Phenylalanine hydroxylation (F)	F	Phe-OH_F
Tryptophan hydroxylation (W)	W	Trp-OH_W
Tyrosine hydroxylation (Y)	Y	Tyr-OH_Y
Valine hydroxylation (V)	V	Val-OH_V

***Note:*** ‘results.json’ contains the prediction results for every position (indexing starts at 1) in every sequence in json format. Each prediction is labeled as <protein uid_site_ptm_type: prediction score>. ‘correct_predictions.csv’ contains only predictions with a prediction score >= 0.5, which are defined as the predicted PTMs. The prediction score ranges from 0 to 1, and higher scores indicate a higher predicted probability for PTM occurrence. Details about the PTM type abbreviations can be found in Table 1.

**CRITICAL:** The above steps illustrate the process of running predictions. All operations in this protocol, including PTM prediction, interpretation and mutation effect inspection, are required to be performed under the MIND directory.

***Note:*** MIND-S allows batch predictions on multiple proteins simultaneously. This can be done by simply including all protein sequences of interest in the input fasta file.

### Identification of important amino acid for PTM predictions via interpretation module

**Timing: 5 min**

This step will compute saliency scores to denote the significance of flanking amino acids in the PTM prediction using the integrated gradients method, where a high saliency score of an amino acid indicates a high predicted importance for the PTM.

4. Determine the PTM of interest.
  a. From ‘correct_predictions.csv’ choose the PTM of interest {uid: Q5S007, site: 1444, PTM type: Phos_ST}
5. Run the following code to make interpretations.

>python predict_saliency.py \

> --inter \

> --pretrain_name saved_model/MIND_fifteenfold \

> --data_path sample/Q5S007.fa \

> --res_path result \

> --site 1444

> --ptm_type Phos_ST

***Note:*** Similar to the previous step, pretrain_name specifies the model used; data_path is the path to the protein fasta file and res_path is the path to the output folder. site and ptm_type refer to the position of the PTM interested within the protein sequence (indexing starts at 1) and the PTM type. inter informs the model to execute in interpretation mode. We allow batch interpretation on multiple PTMs within a protein sequence as well. Users can provide the PTM sites and their corresponding types as comma-separated lists as shown below. This step will also generate a figure visualizing the interpretation scores.

>python predict_saliency.py \

> --inter \

> --pretrain_name saved_model/MIND_fifteenfold \

> --data_path sample/Q5S007.fa \

> --res_path result \

> --site 6,1269,935,1489 \

> --ptm_type Palm_C,glyco_N,Phos_ST,glyco_N

6. Check the saliency scores figure generated in the result directory (Figure 2).

***Note:*** The interpretation module will generate a figure showing the saliency scores of 10 flanking amino acids on both sides of the PTM site. In the batch interpretation case, one figure will be generated for each PTM. Peaks in the saliency score figure indicate that the amino acids at these positions are important for the prediction.

**CRITICAL:** For a specific PTM predicted by MIND-S in the previous step, users can leverage the interpretation module in MIND-S to investigate the contribution of adjacent amino acids to that PTM (Though it can be extended to every amino acid in the protein, we found in practice usually the local amino acids are more important).

***Note:*** The supported PTM types and their abbreviations are shown in Table 1.

### Evaluation of mutation impact on the PTM landscape

**Timing: 5 min**

This step examines altered PTM landscape introduced by mutations on the protein and generates files highlighting the mutations that altered the PTMs.

7. Run the following to examine the SNP effect:

>python PTMSNP.py \

>--pretrain_name saved_model/MIND_fifteenfold \

>--data_path sample/Q5S007.fa \

>--res_path result \

>--snp R_1441_C \

>--n_fold 15

***Note:*** Similarly, we use pretrain_name to specify the model to perform the predictions and n_fold to specify a 15-fold bootstrap method. We provide the path to the fasta file with data_path, and the result will be stored in the res_path. snp indicates the mutation of interest in the format of “WT_site_MUT”, where WT is the wildtype amino acid; site is the location of the amino acid; MUT is the mutant amino acid. In our example, R is the wild-type amino acid, 1441 is the site of the mutation, and C is the mutant amino acid. Two files ‘Q5S007.json’ and ‘Q5S007_R1441C.json’ for the wild-type and mutant prediction results will be generated along with a ‘Q5S007_R1441C.csv’ that summarizes the change in prediction scores. We allow batch evaluation on multiple mutation within a protein sequence as well. Users can provide the mutations in the same format as comma-separated lists as shown below.

8. Run the following to visualize the altered PTM landscape by step 1:

**Figure 3:**
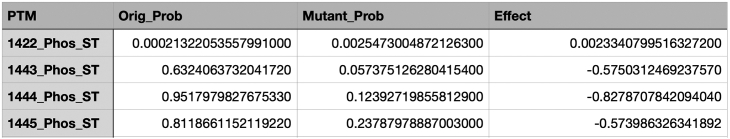
Example Outputs for PTM Prediction with SNP Screenshots from the wild-type and mutant prediction results ‘Q5S007_R1441C.csv’. PTM column represents the specific PTM in the format of <PTMsite_PTMtype>. Prediction scores of the original protein sequence and the mutant protein sequence are shown under the column Orig_Prob and Mutant_Prob respectively. The difference between the mutant sequence and wild-type sequence is shown in the Effect column.

>python ptmfigure.py \

>--orig_path result/Q5S007.json \

>--mutant_path result/Q5S007_R1441C.json \

>--res_path result

***Note:*** Here we specify the wild-type predictions (orig_path) and mutant predictions (mutant_path) to create a csv file summarizing the predictions as well as a figure highlighting the significant difference between the probabilities of the wild-type and the mutant (Figure 3). The resulting files will be stored in res_path.

**Figure 4:**
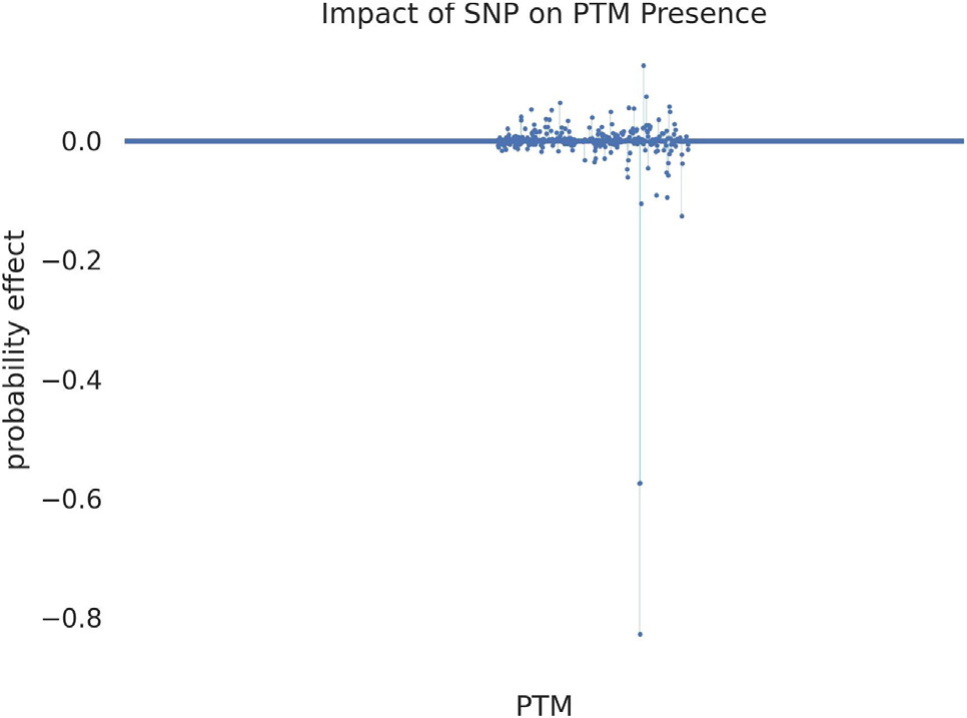
Scatter Plot of SNP effect on PTM expression Each point in the figure represents a unique PTM. X-axis represents the protein sequence from N-terminal to C-terminal. The y-axis represents the difference between mutant expression probability and wild-type expression probability. Positive y-values indicate a higher probability in mutant while lower in wildtype, and negative y-values indicate a lower probability in mutant while higher in wildtype. PTMs that are highly impacted by the mutation are readily visible in this figure.

9. Examine the final figure stored in results directory, where SNPs with high impact will stand out (Figure 4).

## Expected outcomes

A list of files and figures will be produced from running the protocol: “results.josn” is a JSON file containing prediction results of all PTM types on all targeted amino acids. PTM information and prediction scores are included; “correct_predictions.csv” is a csv file containing only positive prediction results (prediction score > 0.5). PTM information and prediction scores are included; Interpretation figure will be produced as a line plot showing the saliency scores of amino acids surrounding the PTM site of interest (Figure 2); two JSON files containing the predictions from the wild-type and mutant protein sequences will be generated (Figure 3); A scatter plot showing the mutation effect on every PTM on the protein (Figure 4).

## Limitations

We suggest users be mindful in interpreting the prediction scores. As per PTM, the prediction problem is a binary classification where we used 1 to indicate the positive PTM and 0 for the negative PTM. A score close to 1 indicates that the site is likely to have the PTM and a score close to 0 indicates that the site is unlikely to have the PTM. For evaluating the mutation effect, we suggest only considering the altered PTM with a prediction score difference greater than 0.2 to minimize the impact of noise.

In the interpretation step, experimentally identified PTMs can also be used as input. However, we recommend verifying if the PTM is predicted by the MIND-S program, since the interpretation is strongly associated with the model’s specific predictions. The interpretation module is best used for experimental PTMs that are also predicted by MIND-S.

## Troubleshooting

### Problem 1

During installation, an error may occur depending on the local environment. (Set up the environment for running the program step 6).

>ModuleNotFoundError: No module named 'module'

### Potential solution

Create a Python virtual environment and install the required modules according to the environment setup steps.

>python -m venv venv

>source venv/bin/activate

>python -m pip install –upgrade pip

>pip install -r requirement

## Resource availability

### Lead contact

Further information and requests for resources and reagents should be directed to and will be fulfilled by the lead contact, Peipei Ping (pping38@g.ucla.edu).

### Materials availability

This study did not generate new unique reagents.

## Data Availability

The Code and data utilized during this protocol are available at Zenodo: https://doi.org/10.5281/zenodo.8393338.
