# Supplementary material for: Protocol for the prediction, interpretation, and mutation evaluation of post-translational modification using MIND-S
Source: STAR Protoc. 2023 Nov 18;4(4):102682. doi: 10.1016/j.xpro.2023.102682 (PMC10694567; doi:10.1016/j.xpro.2023.102682)
Supplement: Document S1. Figures S1 [file mmc1.pdf]

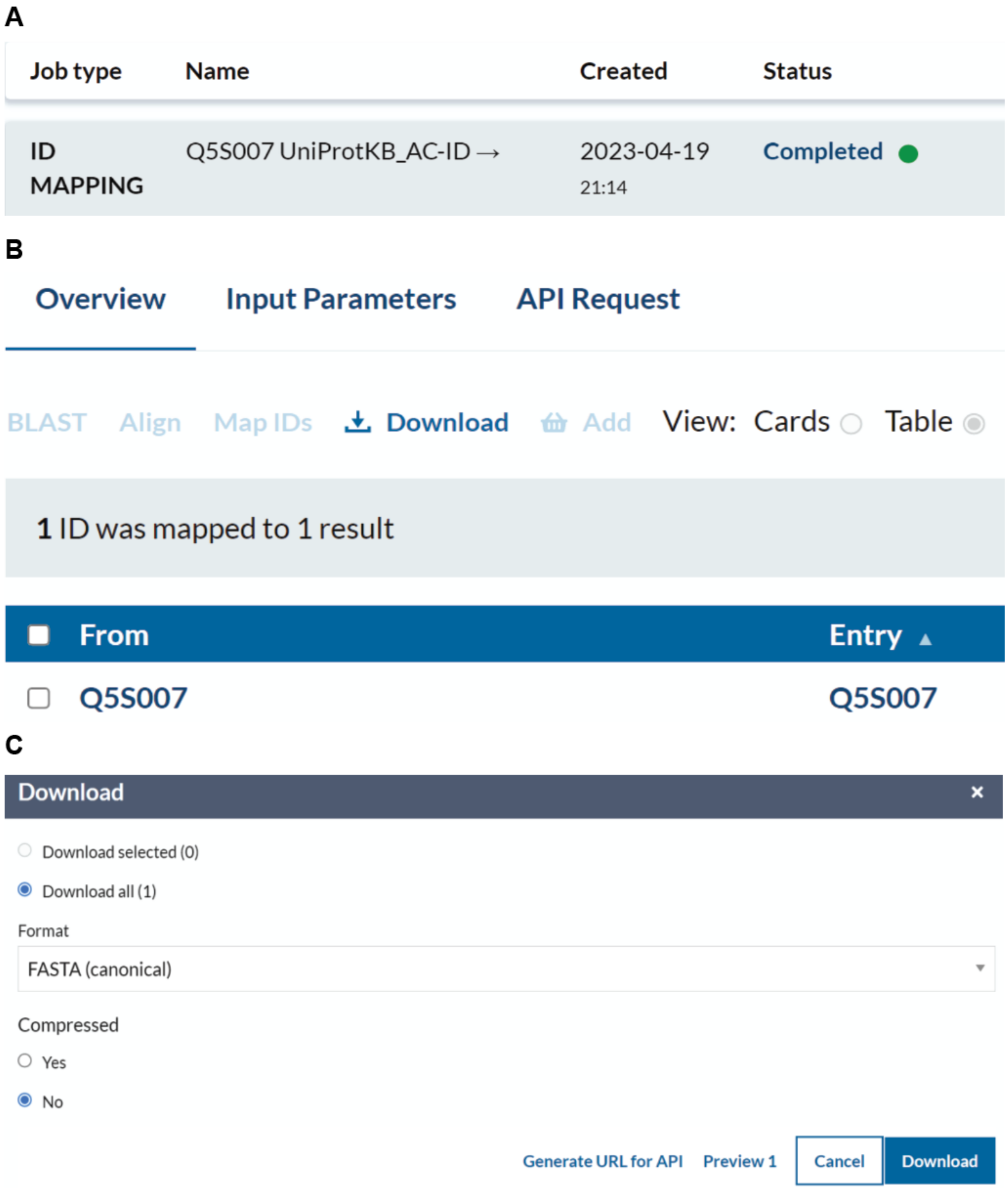

Figure S1. Demonstration of downloading protein fasta files from Uniprot, Related to "Before you begin", Step 1
(A) Enter UniProt ID (e.g. Q5S007) into the input field, click on 'Map IDs', and wait for the job to complete.

(B) When the search result shows up, select 'Download.'

(C) Select 'FASTA (canonical)' under 'Format' and 'No' under 'Compressed' to download the sequence.
